# No Positive Association between Vitamin D Level and Immune Responses to Hepatitis B and *Streptococcus pneumoniae* Vaccination in HIV-Infected Adults

**DOI:** 10.1371/journal.pone.0168640

**Published:** 2016-12-15

**Authors:** Jean-Paul Viard, Alex Assuied, Yves Lévy, Jean-Claude Souberbielle, Rodolphe Thiébaut, Fabrice Carrat, Geneviève Chêne, Odile Launay, Laura Richert

**Affiliations:** 1 APHP, Centre de diagnostic et de Thérapeutique, Hôtel-Dieu, Paris, France; 2 EA 7327, Faculté de Médecine Paris Descartes, Paris, France; 3 INSERM, ISPED, Centre INSERM U1219, Bordeaux, France; 4 Université Bordeaux, ISPED, Centre INSERM U1219, Bordeaux, France; 5 Equipe 16, INSERM U955, Créteil, France; 6 Faculté de médecine, Université Paris Est, Créteil, France; 7 Vaccine Research Institute (VRI), Créteil, France; 8 Service d'immunologie clinique et maladies infectieuses, AP-HP, Hôpital H. Mondor A. Chenevier, Créteil, France; 9 Service d’explorations fonctionnelles, Hôpital Necker, APHP, Paris France; 10 CHU de Bordeaux, Pôle de santé publique, Bordeaux, France; 11 INRIA SISTM, Talence, France; 12 INSERM U1136, Paris, France; 13 APHP, Saint Antoine Hospital, Paris, France; 14 INSERM, CIC 1417, F-CRIN, I–REIVAC, Paris, France; 15 Université Paris Descartes, Sorbonne Paris Cité, Paris, France; 16 APHP, Cochin Hospital, CIC Cochin Pasteur, Paris, France; Public Health England, UNITED KINGDOM

## Abstract

**Objective:**

To assess whether higher 25-hydroxyvitamin D (25OHD) levels are associated with subsequent better immune responses to hepatitis B and *Streptococcus pneumoniae* vaccination in HIV-infected patients.

**Methods:**

25OHD was measured on stored baseline plasma samples from two randomized vaccine trials in HIV-infected adults: the ANRS HB03 VIHVAC B trial and an immunological sub-study of the ANRS 114-PNEUMOVAC trial. In ANRS HB03 VIHVAC B, participants received three or four doses of recombinant HBV vaccine strategies. Anti-HBs IgG titers were measured four weeks after the last injection. Associations between baseline 25OHD levels and ordered IgG response categories were analyzed in multivariable proportional odds models. In the ANRS 114-PNEUMOVAC sub-study, two strategies of pneumococcal vaccination were tested, cellular immune responses were measured at repeated time points, and IgG responses four weeks after the last vaccine injection. Exploratory statistical analyses were performed on this sub-study data set.

**Results:**

Three hundred and thirty-nine ANRS HB03 VIHVAC B and 25 ANRS 114-PNEUMOVAC sub-study participants were included in the analyses. Median age in each of the two studies was 43 years, 68% were male, and 77–92% on antiretroviral treatment. Median 25OHD level was 18 ng/mL (IQR: 12–25) and 24 ng/mL (IQR: 13–32) in the two trial populations, respectively. In the multivariable model, there was no significant association between baseline 25OHD level and vaccine responses in ANRS HB03 VIHVAC B (proportional odds ratio 0.83 per 10 ng/mL 25OHD increase; 95% confidence interval 0.65–1.07, p = 0.14). Exploratory analyses of ANRS 114-PNEUMOVAC showed consistent results.

**Conclusion:**

This study does not support a positive association between 25OHD and immune responses to hepatitis B or pneumococcal vaccination in HIV-infected patients.

## Introduction

Vitamin D is increasingly recognized as a regulator of immune functions [1]. Innate and adaptive immune cells harbour the vitamin D receptor, and vitamin D has been shown to play a role in the initial activation of naïve T-cells [2]. The vitamin D receptor is involved in regulation of gene expression and has direct transcription activity, playing a role in physiological processes such as cell proliferation and differentiation. Moreover, antigen-presenting cells as well as lymphocytes express the 1-α hydroxylase to convert serum 25-hydroxyvitamin D (25OHD) to its active form (1,25-dihydroxvitamine D), resulting in local intra- and paracrine actions. Studying the role of vitamin D on the functions of the immune system is an active field of research, and current knowledge indicates that vitamin D has various immunomodulatory effects, including actions on the innate immune system and orientating T-cellular immune responses towards a Th2 phenotype [3].

In epidemiological studies in the general population, 25OHD deficiency has been associated with increased susceptibility to infections, particularly upper respiratory tract infections and tuberculosis, and a meta-analysis suggested that vitamin D supplementation offers some protection against respiratory tract infections [4]. In HIV-infected populations, 25OHD deficiency has been associated with unfavourable clinical evolution in both ART-naïve and ART-treated patients [5,6], possibly through its effects on immunity.

In consequence, it has been hypothesized that vitamin D levels could have an impact on immune responses to vaccines, mediated by its effects on immune homeostasis and immunomodulation. Indeed, in mice, administration of vitamin D as an adjuvant during vaccination has shown to increase vaccine-induced immune responses (reviewed in [3]). In humans, several studies have shown that vitamin D level or supplementation does not influence the response to influenza vaccination, including in HIV-infected persons [7–13]. However, few data are available concerning the relationship between vitamin D level and vaccine responses against other pathogens, such as hepatitis B or *Streptococcus pneumoniae* [14–16]. In a retrospective study in patients with chronic kidney disease, vitamin D deficiency was found to be negatively associated with seroconversion after hepatitis B vaccine [14].

Correction of 25OHD deficiency is a simple and harmless intervention. Demonstration of an association between 25OHD deficiency and poor vaccine responses could thus lead to supplementation trials aiming at improving vaccine responses, particularly in immuno-compromised populations.

We therefore examined whether higher baseline 25OHD levels were associated with better responses to hepatitis B or *S*. *pneumoniae* vaccination in two vaccine trials in HIV-infected adults.

## Materials and Methods

This study used data from the randomized ANRS HB03 VIHVAC B and ANRS 114-PNEUMOVAC clinical trials in HIV-infected adults. The ANRS HB03 VIHVAC B trial (clinicaltrials.gov NCT00480792) tested the immunogenicity of three or four doses of recombinant HBV vaccine (three intramuscular 20 μg injections at W0, W4 and W24; four intramuscular 40 μg injections, or four intradermal 4 μg injections at W0, W4, W8 and W24). The ANRS 114-PNEUMOVAC trial (clinicaltrials.gov NCT00148824) evaluated two strategies of pneumococcal vaccination (7-valent conjugate vaccine at W0 followed by 23-valent polysaccharide vaccine at W4 vs. one dose of the polysaccharide vaccine at W4 without any vaccination at W0).

Each trial was approved by the relevant ethics committee (“CPP Ile de France 3” and “CCPPRB Créteil-Henri Mondor” for ANRS HB03 VIHVAC B and ANRS 114-PNEUMOVAC, respectively) and was conducted in accordance with the Declaration of Helsinki. All participants provided written informed consent. Methods and primary results of the trials have been reported previously [17,18].

For the current study, participants from each of the two trials were eligible if they had a stored baseline plasma sample (from either trial screening or W0 visit before vaccination), available antibody data, and if they had given informed consent for further use of their samples. For the ANRS 114-PNEUMOVAC trial, an extra eligibility criterion was consent to an immunological sub-study that assessed cellular CD4 T-cell responses in addition to antibody measurements [19]. 25OHD was measured in the stored plasma samples from all eligible participants of both trials in a single centralized laboratory using the DiaSorin radioimmunoassay technique [20], which was calibrated against the NIST (National Institutes of Standards and Technology) standard. In descriptive analyses, 25OHD level was categorized as sufficient (≥ 30 ng/mL), insufficient (10–29 ng/mL) and deficient (<10 ng/mL).

In the ANRS HB03 VIHVAC B trial, anti-HBs antibody responses to vaccination were measured at W28. As defined *a priori* in the study protocol, antibody responses levels were treated as an ordered categorical endpoint in the statistical analyses, using the following response categories: hepatitis B vaccine non responders (anti-HBs ≤ 10 mIU/mL), low responders (10 mIU/mL < anti-HBs ≤ 100 mIU/mL) and high responders (anti-HBs > 100 mIU/mL). The cut-off of 10 mIU/mL reflects an anti-HBs titer level consistent with seroprotection, while the 100 mIU/mL cut-off is considered to confer higher and long-term protection against infection [17, 21, 22]. Associations between baseline 25OHD level, treated as a quantitative variable, and ordered antibody response levels were analyzed in proportional odds models in the ANRS HB03 VIHVAC B trial, as its sample size was sufficiently large. This model is a generalization of the logistic model for an ordered categorical response variable instead of a binary variable, and estimated proportional odds ratios reflect the odds of being in any higher antibody response category [23]. The randomized arms of the trial were pooled for these analyses but multivariable models were used, adjusting for the trial arms and for the following clinical, behavioural and socio-demographic determinants of vaccine responses reported in the primary analyses of the trial [17]: sex, age, active smoking status, baseline CD4 count and plasma HIV-1 RNA. Adequacy of the proportional odds assumption was checked using a score test. We further performed sensitivity analyses, using a binary anti-HBs antibody response definition in a logistic regression model, with a cut-off of 10 mIU/mL as done in the primary endpoint analysis of the original trial report [17].

Power calculations under the simplified and conservative assumption of a binary 25OHD explanatory variable (dichotomized by the median) indicated that the ANRS HB03 VIHVAC B dataset would provide at least 80% power to detect a univariable proportional odds ratio of 1.7 with a two-sided type 1 error rate of 5%.

In the ANRS 114-PNEUMOVAC sub-study anti-pneumococcal IgG antibody measurements were performed at W8, and cellular CD4 T-cell responses to the diphtheria-derived carrier protein CRM_197_ of the conjugate anti-pneumococcal vaccine were measured at W0, W4 and W24. Antibody levels to each of the 7 serotypes shared by the 7-valent conjugate and 23-valent polysaccharide vaccine (serotypes 4, 6B, 9V, 14, 18C, 19F, and 23F) were determined. Per participant and serotype, presence of antibody response was defined as participant who experienced both a 2-fold post-vaccination antibody increase and a level of serotype specific IgG of ≥1 mg/mL [19]. IgG response to vaccination at W8 was summarized according to the number of pneumococcal serotypes (0–2, 3–4, 5–7) a participant developed a response to. Cellular responses were analyzed in the group having received the 7-valent conjugate vaccine prime containing the diphtheria-derived carrier protein CRM_197,_ and included lymphocyte proliferative responses (expressed as stimulation index, i.e. counts per minute in stimulated vs. unstimulated cultures) and Th1-associated T-cell cytokine responses (production of interleukin-2 or interferon-gamma, measured in supernatants) after stimulation of peripheral blood mononuclear cells with the conjugated carrier protein CRM_197_ or with diphtheria toxin. Details of the laboratory methods for these assays have been previously described [19].

The associations between baseline 25OHD and immune responses in the ANRS 114-PNEUMOVAC sub-study, which had a small sample size (n = 25) available for the present analyses, were assessed by non-parametric tests (Kruskal-Wallis test and Spearman rank correlations) instead of regression models. Acknowledging the limited statistical power of the small sample size, we used this independent dataset for exploratory analyses to assess whether the overall signals were consistent with the conclusions from the ANRS HB03 VIHVAC B trial’s analyses. We furthermore explored potential associations between baseline 25OHD level and the cellular immune response measurements available in the ANRS 114-PNEUMOVAC sub-study. Spearman correlations between baseline 25OHD and the change in cellular responses after vaccination were assessed in the group having received the 7-valent conjugate vaccine prime containing the diphtheria-derived carrier protein CRM_197_ at W0 (n = 11). For these analyses, change in a given cellular response was calculated as the individual difference in the level of the response between W0 to W4 (the peak time point of cellular responses [19]), i.e. the W0 value was subtracted from the W4 value, for the log stimulation index and for interferon-γ and interleukin-2 levels in supernatants. Given the limited statistical power, search for any signals focussed on the direction of effects more than on p-values.

Data from each trial were analyzed separately, based on available data. Analyses were performed using SAS software, version 9.3 (SAS Institute, Cary, North Carolina).

## Results

Out of 426 participants randomized and vaccinated in the ANRS HB03 VIHVAC B trial, 339 fulfilled the eligibility criteria of the present study and were included in the analyses. Their baseline characteristics are summarized in Table 1 and there were no clinically relevant differences with the baseline characteristics of the entire trial population [17]. The median baseline 25OHD level was 18 ng/mL (IQR: 12–25), with 17% of patients having sufficient 25OHD levels (≥ 30 ng/mL), 70% having insufficient levels (10–29 ng/mL) and 13% having deficiency (< 10 ng/mL). A higher proportion of participants living in Northern France had 25OHD levels below 30ng/mL compared to participants living in Southern France (88% vs. 72%, p<0.001). Likewise, the proportion of 25OHD levels below 30ng/mL was higher in participants with a baseline sample collected in winter/spring compared to participants whose sample was collected in summer/fall (94% vs. 75%, p<0.001). There was no significant association between baseline body mass index (BMI) and 25OHD levels.

**Table 1:** Baseline characteristics of the study populations in the ANRS HB03 VIHVAC B trial and the ANRS 114-PNEUMOVAC sub-study.

	ANRS HB03 VIHVAC B trial (n = 339)	ANRS 114-PNEUMOVAC sub-study (n = 25)
Age (years), median, IQR	43 (36–49)	43 (36–50)
Male sex, n, %	229 (68)	17 (68)
On antiretroviral treatment, n, %	261 (77)	23 (92)
CD4 count (cells/mm^3^), median, IQR	508 (401–658)	375 (302–405)
HIV-1 RNA level < 500 cp/mL, n, %	295 (87)	20 (80)
CDC category C, n, %	46 (14)	13 (52)
Active smoking, n, %	109 (32)	9 (36)
25OHD level (ng/mL), median, IQR	18 (12–25)	24 (13–32)
25OHD status, n, %		
Sufficiency (≥ 30 ng/mL)	57 (17)	8 (32)
Insufficiency (10–29 ng/mL)	236 (70)	14 (56)
Deficiency (< 10 ng/mL)	46 (13)	3 (12)

There was no statistically significant association between baseline 25OHD status (sufficiency, insufficiency and deficiency) and vaccine response categories (Table 2, Chi-squared test p = 0.09).

**Table 2:** Association between baseline 25OH Vitamin D status and response to hepatitis B vaccination in the ANRS HB03 VIHVAC B trial (n = 339).

	Vaccine non responders (n = 52)	Vaccine low responders (n = 61)	Vaccine high responders (n = 226)	*P*-value
Baseline 25OHD status, n, %				0.09
Sufficiency (≥ 30 ng/mL)	11 (21%)	14 (23%)	32 (14%)	
Insufficiency (10–29 ng/mL)	39 (75%)	39 (64%)	158 (70%)	
Deficiency (< 10 ng/mL)	2 (4%)	8 (13%)	36 (16%)	

Vaccine non responders: anti-HBs ≤ 10 mIU/mL; vaccine low responders: 10 mIU/mL < anti-HBs ≤ 100 mIU/mL; vaccine high responders: anti-HBs > 100 mIU/mL

Baseline 25OHD levels assessed as a continuous variable also did not significantly differ between the antibody response groups (see Table 3 for median 25OHD levels per antibody response group, Kruskal Wallis test p = 0.12).

**Table 3:** Association between baseline 25OH Vitamin D level and antibody response to hepatitis B vaccination in the ANRS HB03 VIHVAC B trial (proportional logistic regression model, multivariable analysis, n = 333 with non missing covariates).

	Descriptive analyses			Proportional odds model	
	Vaccine non responders (n = 52)	Vaccine low responders (n = 61)	Vaccine high responders (n = 220)	pOR (95%CI) for higher response to vaccination	*P*-value
Baseline 25OHD (ng/mL), median, IQR (pOR per 10 ng/mL increase)	20 (15–27)	17 (12–28)	18 (11–24)	0.83 (0.65–1.07)	0.14
Vaccine dose group, n, %					<0.0001
20 **μ**g X 3 IM	28 (54%)	24 (39%)	56 (25%)	1 (ref.)	
40 **μ**g X 4 IM	3 (6%)	9 (15%)	96 (44%)	9.32 (4.41–19.70)	
4 **μ**g X 4 ID	21 (40%)	28 (46%)	68 (31%)	1.41 (0.82–2.43)	
Sex, n, %					0.01
Male	45 (87%)	46 (75%)	134 (61%)	1 (ref.)	
Female	7 (13%)	15 (25%)	86 (39%)	2.17 (1.20–3.93)	
Age (years), median, IQR (pOR per 10 year increase)	44 (38–50)	42 (35–50)	42 (36–49)	0.80 (0.60–1.06)	0.12
Acitve smoking status, n, %					<0.0001
Non smoker	20 (38%)	36 (59%)	168 (76%)	1 (ref.)	
Smoker	32 (62%)	25 (41%)	52 (24%)	0.28 (0.16–0.47)	
Baseline CD4 count (cells/mm^3^), median, IQR (pOR per 100 cells/mm^3^ increase)	488 (394–575)	501 (385–694)	514 (405–658)	1.13 (1.01–1.27)	0.03
Baseline plasma HIV-1 RNA, n, %					0.054
< 50 copies /mL	36 (69%)	44 (72%)	188 (85%)	1 (ref.)	
≥ 50 copies/mL	16 (31%)	17 (28%)	32 (15%)	0.55 (0.30–1.01)	

IQR: interquartile range; pOR: proportional odds ratio; 95% CI: 95% confidence interval; IM: intramuscular; ID: intradermal

Vaccine non responders: anti-HBs antibody titer ≤ 10 mIU/mL; vaccine low responders: 10 mIU/mL < anti-HBs antibody titer ≤ 100 mIU/mL; vaccine high responders: anti-HBs antibody titer > 100 mIU/mL

The univariable regression analysis indicated a marginally significant association between lower 25OHD levels and better vaccine response (proportional odds ratio of having a better antibody response category, per 10 ng/mL increase in 25OHD: 0.78 [95% confidence interval: 0.62–0.98, P = 0.03). In multivariable analyses (Table 3), taking into account all determinants associated with vaccine response in the original trial report [16], no significant association was found between baseline 25OHD level and antibody response category (proportional odds ratio: 0.83 per 10 ng/mL increase of 25OHD, 95% confidence interval: 0.65–1.07, p = 0.14). Sensitivity analyses, using a binary anti-HBs antibody response definition in a logistic regression model, showed consistent conclusions regarding the absence of an association in the multivariable model. We further tested for an interaction between baseline 25OHD and vaccine dose group on antibody responses in the multivariable proportional odds model and found no significant interaction.

Among the 60 ANRS 114-PNEUMOVAC sub-study participants, 25 could be included in the present analyses. Their baseline characteristics are shown in Table 1. Median baseline 25OHD level was 24 ng/mL (IQR: 13–32), with 32% of patients having sufficient levels, 56% having insufficient levels and 12% having deficiency.

At W8, 10 participants (40%) had antibody responses to 0–2 serotypes, 5 (20%) to 3–4 serotypes, and 10 (40%) to 5–7 serotypes. In the participants having received the 7-valent conjugate vaccine prime containing the diphtheria-derived carrier protein CRM_197_ (n = 11), CD4 responses at W4 were as follows: median log stimulation index after DT stimulation 0.5 (IQR 0.3–1.8), median log stimulation index after CRM_197_ stimulation 1.0 (IQR 0.3–1.7), median interferon-γ level in supernatants after CRM_197_ stimulation 65 pg/mL (IQR 0–811), and median interleukin-2 level in supernatants after CRM_197_ stimulation 8 pg/mL (IQR 2–68).

Median baseline 25OHD levels were 19 ng/mL (IQR 13–32), 11 ng/mL (IQR 11–13), and 28 ng/mL (IQR 24–36) in the groups with 0–2, 3–4, and 5–7 serotype responses, respectively (Kruskal-Wallis test, p = 0.20). Spearman correlations between baseline 25OHD and the change in T-cell responses from W0 to W4 in the group having received the 7-valent conjugate vaccine prime containing the diphtheria-derived carrier protein CRM_197_ at W0 (n = 11) are summarized in Table 4. Correlations with baseline 25OHD were close to zero (r < │0.1│) for the change in proliferation responses, assessed as stimulation index after stimulation by diphtheria toxin and CRM_197_, respectively (Table 4). Slightly to moderately negative coefficients were observed between baseline 25OHD and the W0 to W4 change in interferon-gamma (r = -0.16, p = 0.66) and interleukin-2 production (r = -0.40, p = 0.26), respectively, after CRM_197_ stimulation.

**Table 4:** Correlations between baseline 25OHD level and change in cellular immune responses between W0 and W4 in the group having received the 7-valent conjugate vaccine prime containing the diphtheria-derived carrier protein CRM_197_ at W0, ANRS-114 PNEUMOVAC sub-study (n = 11).

Correlation between baseline 250HD level and given variable	Spearman r	p
W4 change in log stimulation index after DT stimulation	0.07	0.84
W4 change in log stimulation index after CRM_197_ stimulation	-0.04	0.90
W4 change in interferon-γ level in supernatants after CRM_197_ stimulation	-0.16	0.67
W4 change in interleukin-2 level in supernatants after CRM_197_ stimulation	-0.40	0.26

CRM_197:_ diphtheria derived carrier protein; DT: diphtheria toxin; W: week

Stimulations were done on peripheral blood mononuclear cells sampled at W0 and W4. W4 change calculated as difference between W4 and W0 values.

## Discussion

We used data from a large clinical trial of hepatitis B vaccination to assess whether higher baseline 25OHD levels were associated with better subsequent antibody responses to vaccine in HIV-infected adults and found no evidence for such an association. Exploratory analyses of a second independent, albeit smaller, dataset from a pneumococcal vaccine trial were consistent with this finding.

Our results might not be generalized to the general population because the HIV status of the patients may have overwhelmed a possible effect of vitamin D on immune responses. However, most patients in the study had controlled HIV infection and preserved CD4 cell counts [17,18]. Previous studies on 25OHD levels and responses to influenza vaccination also showed no associations, and this held true both in the general and HIV-infected populations [7–13]. Our own results do not support a positive association between 25OHD and antibody response to hepatitis B vaccine in HIV-infected persons.

Vitamin D deficiency favours inflammation and immune activation [1], which may have deleterious effects in conditions with pathogen-induced immune-mediated abnormalities, and vitamin D supplementation trials have shown favourable effects on inflammation and immune cell activation in persons with tuberculosis or HIV infection [24,25]. Vitamin D deficiency could therefore be seen as a potentially modifiable risk factor for inflammation, and supplementation trials are likely to be pursued in these settings, with vitamin D supplementation now being widely used in patients with chronic diseases, including HIV infection. A concern in this perspective is that the known immunomodulatory effects of vitamin D may actually be deleterious for vaccine responses. Vitamin D has anti-inflammatory properties, down regulates T-cell activation, B-cell differentiation, and dendritic cell functions, and stimulates the generation of regulatory T-cells [1]. It is seen as modulating immune responses towards less inflammation and more tolerance, which might arguably hamper the induction of vaccine responses. A small study in HIV-uninfected, immuno-compromised asplenic persons specifically tested the hypothesis whether higher 25OHD levels inhibit vaccine responses. No clear significant associations between 25OHD levels and lower antibody responses to *S*. *pneumoniae*, *N*. *meningitidis* or *H*. *influenzae* vaccination were found, although non-significant trends for moderate negative correlations between 25OHD levels and antibody titers were observed for some vaccine responses [16]. Although our univariable results could also be interpreted as a possible signal for such a negative association, this was not statistically significant in the multivariable model of the ANRS HB03 VIHVAC B dataset. However, we can neither exclude limited statistical power nor residual confounding in our multivariable results. Altogether, should any deleterious associations exist they do not seem to be of relevant magnitude and are not consistently observed across studies.

Our study has the advantage of including a relatively large prospective data set of a randomized multicentre trial of hepatitis B vaccination in HIV-infected patients with standardized measurements of 25OHD in a central laboratory. However, the fact that not all participants of the core trial could be included in the present analyses could be a potential limitation. The sample size of the second data set on pneumococcal vaccination is very small and has different immunogenicity endpoints. The analyses of this data set should thus be considered exploratory.

In conclusion, our study in the setting of HIV infection, indicate that 25OHD levels, be they in the higher or lower spectrum, do not have any positive associations with vaccine responses to hepatitis B or *S*. *Pneumonia* vaccination.
